# Increased APOBEC3G and APOBEC3F expression is associated with low viral load and prolonged survival in simian immunodeficiency virus infected rhesus monkeys

**DOI:** 10.1186/1742-4690-8-77

**Published:** 2011-09-28

**Authors:** Bianka Mußil, Ulrike Sauermann, Dirk Motzkus, Christiane Stahl-Hennig, Sieghart Sopper

**Affiliations:** 1 Unit of Infection Biology, German Primate Centre, Goettingen, Germany; 2 Unit of Infection Models, German Primate Centre, Goettingen, Germany; 3 Dept. of Hematology and Oncology, Medical University Innsbruck, Innsbruck, Austria; 4 URM, Institut Pasteur, 28 rue du Dr Roux, 75015 Paris, France

## Abstract

**Background:**

The cytidine deaminases APOBEC3G (A3G) and APOBEC3F (A3F) are innate cellular factors that inhibit replication of a number of viruses, including HIV-1. Since antiviral activity of APOBEC3 has been mainly confirmed by *in vitro *data, we examined their role for disease progression in the SIV/macaque model for AIDS.

**Results:**

We quantified A3G and A3F mRNA in PBMC and leukocyte subsets of uninfected and SIVmac-infected rhesus macaques. Compared with uninfected animals, we found increased A3G and A3F mRNA levels in PBMC, purified CD4+ T-cells and CD14+ monocytes as well as lymph node cells from asymptomatic SIV-infected macaques. APOBEC3 mRNA levels correlated negatively with plasma viral load, and highest amounts of APOBEC3 mRNA were detected in long term non-progressors (LTNPs). During acute viremia, A3G mRNA increased in parallel with MxA, a prototype interferon-stimulated gene indicating a common regulation by the initial interferon response. This association disappeared during the asymptomatic stage.

**Conclusion:**

Our findings suggest a protective effect of APOBEC3 for HIV and SIV *in vivo *and indicate regulation of APOBEC3 by interferon during early infection and by contribution of other, hitherto undefined factors at later disease stages. Elucidating the regulatory mechanisms leading to increased APOBEC3 mRNA levels in LTNPs could help to develop new therapies against HIV.

## Background

Infection with HIV leads to the development of severe immunodeficiency in a widely variable time frame. A small percentage of the HIV-infected individuals, the long term non-progressors (LTNPs) even remain clinically healthy without symptoms for over 15 years [1]. Those differences are thought to result from the interaction of virus and host factors influencing viral replication. Two recently described innate host factors in humans, APOBEC3G (hA3G) and APOBEC3F (hA3F), possess antiretroviral activity and have been shown to restrict HIV-1 replication *in vitro *[2-4]. In the absence of the HIV-1 accessory protein Vif, hA3G and hA3F are incorporated into virus particles and impair retroviral replication by introducing G-to-A hypermutations in the viral genome [3,5,6]. However, Vif counteracts the activity of hA3G and hA3F and prevents their encapsidation into virions by promoting their proteasomal degradation via ubiquitination [7-9]. In addition to the editing-mediated restriction by APOBEC3 deaminases, also other non-enzymatic inhibitory mechanisms have been described, some of which seem to be less susceptible to inhibition by Vif [10,11]. Despite Vif expression, low levels of APOBEC3-mediated cytidine deamination are detectable, indicating that even wild-type HIV-1 can be restricted to some extent by the presence of APOBEC3 proteins [12,13]. Also, higher levels of A3G expression are able to overcome the effects of Vif [3,4,14], suggesting that regulation of A3G expression may represent a novel target for antiretroviral therapy. In this regard, several studies demonstrated regulation of APOBEC3 by interferons or other immune mediators *in vitro *[15-22]. Several lines of evidence indicate that APOBEC3 may indeed have an impact on disease progression in HIV-infected patients. First, a genetic variant of A3G was reported that was associated with steeper CD4 T-cell decline and faster disease progression in HIV-infected African Americans [23]. Furthermore, APOBEC driven G-to-A hypermutations in the viral genome occurring *in vivo *during the early phase of HIV-1 infection also may have an influence on disease progression by facilitating early immune escape [24]. Finally, higher levels of A3G and A3F were documented for HIV-1 infected individuals with lower viral set points [25,26]. This finding however has been challenged by other studies, which did not find a correlation between hA3G and hA3F mRNA levels and viral load [27].

Infection of macaques with simian immunodeficiency viruses (SIV) is currently the best animal model to study HIV infection and AIDS pathogenesis [28,29]. Although experimental infection of rhesus macaques with SIV isolates leads to disease and death in a shorter time-frame compared with HIV infection, a similar variability in disease course with progressors and long term non progressors (LTNP) has also been observed in SIV infection [30,31]. Furthermore, it has been demonstrated that rhesus APOBEC3 enzymes are also able to restrict SIV replication [32] and that they are similarly degraded via Vif-dependent mechanisms [33]. Taken together, the SIV rhesus macaque model for AIDS provides the necessary components to investigate the role of APOBEC3 for disease progression under defined experimental settings. Therefore, we used this model to determine A3G and A3F mRNA levels in different cellular compartments. Levels of A3G and A3F were correlated with viral load and disease progression. In addition, we assessed a possible regulation of APOBEC3 through interferons *in vivo *by transcription analysis of prototype interferon stimulated genes (ISGs).

Our results show significantly increased amounts of A3G and A3F mRNA in SIV-infected asymptomatic macaques with the highest APOBEC3 mRNA levels detected in PBMC, purified CD4+ T-cells and CD14+ monocytes as well as in peripheral lymph nodes of LTNPs. Furthermore, we found an inverse correlation between APOBEC3 mRNA levels and viral load, suggesting a potential role of APOBEC3 in reducing the viral load. Hence, our data in the SIV rhesus macaque model strongly suggest a protective effect of APOBEC3 in the pathogenesis of AIDS. In addition, we found evidence for a differential regulation of APOBEC3 transcription in distinct disease stages.

## Results

### Increased APOBEC3 mRNA levels in asymptomatic SIV-infected rhesus macaques

In order to study the impact of SIV infection on the APOBEC3 transcription, we determined A3G and A3F mRNA levels in 12 uninfected and 53 SIV-infected rhesus macaques. Infected animals were grouped according to their clinical stage. Twenty-nine macaques investigated during the chronic disease stage were clinically asymptomatic, whereas 24 macaques displayed signs of AIDS.

**Figure 1:**
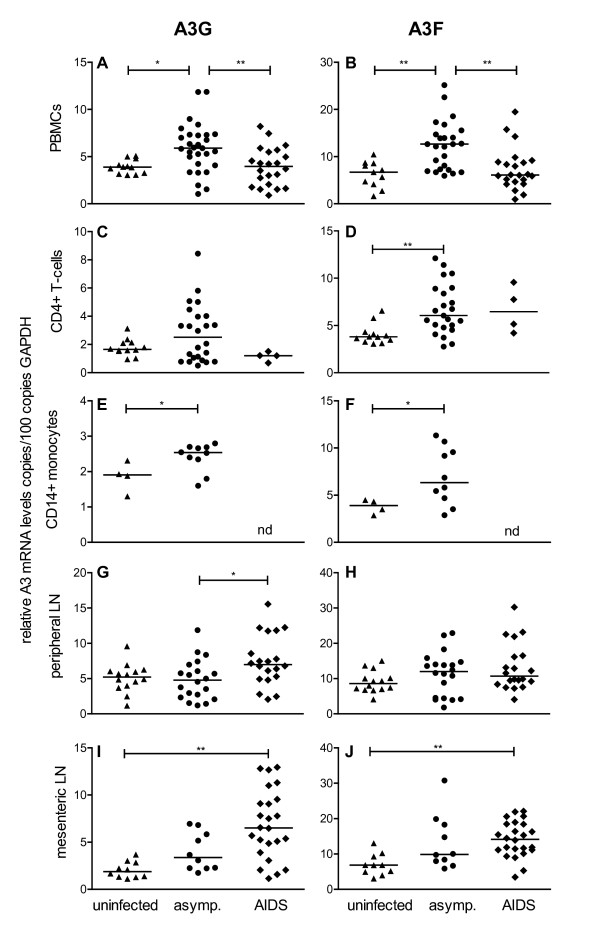
**APOBEC3 mRNA levels in uninfected and SIV-infected macaques**. A3G (left panels) and A3F (right panels) mRNA levels were determined in uninfected (triangles) and SIV-infected animals with (diamonds) or without (circles) AIDS symptoms. Relative APOBEC3 mRNA levels are shown in copy numbers per 100 copies of GAPDH in PBMC (A, B), CD4+ T-cells (C, D), CD14+ monocytes (E, F), lymphocytes from peripheral (G, H) and mesenteric lymph nodes (I, J). Each data point represents one individual animal. Horizontal lines within the clusters are depicting the median. Group comparisons were calculated using either the Kruskal-Wallis test with Dunn's multiple comparison analysis for PBMC, CD4+ T-cells and peripheral lymph nodes or the Mann-Whitney test for CD14+ monocytes (*p < 0.05; **p < 0.001). nd, not determined; asymp., asymptomatic.

Our results show significantly increased levels of A3G and A3F mRNA in peripheral blood mononuclear cells (PBMC) of asymptomatic SIV-infected animals compared with uninfected macaques and animals with AIDS (Figure 1A and 1B). In macaques with AIDS however, A3G and A3F mRNA levels were not significantly different from uninfected controls (Figure 1A and 1B). To further study APOBEC3 levels in potential target cells, we purified CD4+ T-cells and CD14+ monocytes with magnetic beads from PBMC of a subset of animals. Compared with uninfected macaques, significantly increased A3F mRNA levels were found in CD4+ T-cells of SIV-infected asymptomatic animals (Figure 1D). Some asymptomatic SIV-infected animals also showed high A3G mRNA levels compared with uninfected controls, without reaching significance (Figure 1C). Similar to PBMC and CD4+ T-cells, A3G and A3F mRNA levels in CD14+ monocytes were elevated in SIV-infected asymptomatic animals compared with uninfected macaques (Figure 1E and 1F). This cell type could not be investigated in animals with AIDS due to insufficient material. As a representative site of major virus replication, we further quantified A3G and A3F mRNA levels in peripheral and mesenteric lymph nodes. Asymptomatic SIV-infected macaques also showed a similar tendency to higher APOBEC3 mRNA levels compared with uninfected animals, which however failed to reach significance after *post hoc *correction for multiple comparisons (Figure 1G-J). However, contrasting the results from PBMC, animals with AIDS showed even higher A3G mRNA levels in peripheral (Figure 1G) and mesenteric lymph nodes (Figure 1I). Similar results were obtained for A3F mRNA in mesenteric lymph nodes of AIDS animals (Figure 1J).

### Negative correlation of A3G and A3F mRNA with viral load and disease progression

**Figure 2:**
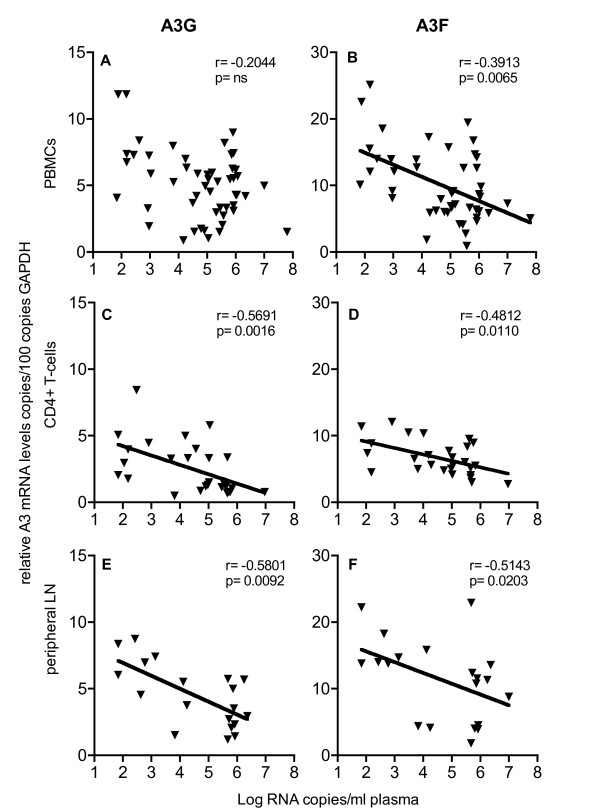
**Relationship of APOBEC3 mRNA levels and plasma viral load**. A3G (left panels) or A3F (right panels) mRNA levels were correlated with plasma viral load. Relative APOBEC3 mRNA levels are shown in copy numbers per 100 copies of GAPDH in PBMC (A, B), CD4+ T-cells (C, D) and peripheral lymph nodes (E, F). Viral load is depicted as log-transformed RNA copies per millilitre (ml) plasma. r, Spearman's correlation coefficient; line shows nonlinear regression; p, P value; ns, not significant.

The high variation of APOBEC3 levels in SIV-infected asymptomatic animals prompted us to look into a possible association with disease progression. Plasma viral load represents the most common early predictor for disease progression in HIV-infected patients [34,35]. A comparable relationship between plasma viral load and SIV infection has also been described in SIV-infected rhesus monkeys [36]. Therefore, we correlated A3G and A3F mRNA levels with plasma viral load. Our results showed negative correlations between plasma viral load and A3G and A3F mRNA levels in both total PBMC and purified CD4+ T-cells (Figure 2A-D), which however was not significant for A3G in the PBMC (Figure 2A). For the peripheral lymph nodes, a negative correlation between A3G and A3F mRNA levels and viral load was seen after exclusion of symptomatic animals with AIDS (Figure 2E and 2F). At necropsy sufficient material was available to directly determine cell associated viral load in lymphoid tissue, correlating well with viral RNA levels in plasma (additional file 1). Cell associated viral load in lymph node cells was inversely correlated with local A3G mRNA levels (additional file 1).

**Figure 3:**
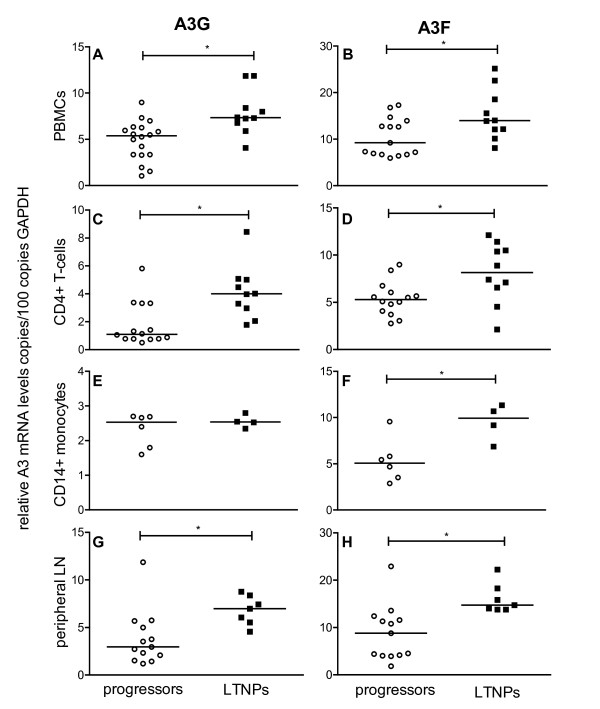
**APOBEC3 mRNA levels in SIV-infected animals with different disease progression**. A3G (left panels) and A3F (right panels) mRNA levels were determined in SIV-infected progressors (open circles) or LTNPs (squares). Relative APOBEC3 mRNA levels are shown in copy numbers per 100 copies of GAPDH in PBMC (A, B), CD4+ T-cells (C, D), CD14+ monocytes (E, F) and lymphocytes from peripheral lymph nodes (G, H). Each data point represents one individual animal. Horizontal lines within the clusters are depicting the median. Group differences were calculated using the Mann-Whitney test (*p < 0.05).

This negative correlation between A3G and A3F expression and viral load found in PBMC, CD4+ T-cells and peripheral lymph nodes suggests an association between APOBEC3 expression and disease progression. Therefore, we divided the SIV-infected asymptomatic macaques into distinct groups according to their survival time, *i *) Progressors with a viral load above 10^4 ^copies per ml plasma being asymptomatic without immunodeficiency when investigated, but featuring a progressive disease course to AIDS within three years post infection, and *ii *) LTNPs representing asymptomatic animals, that had survived for more than three years post infection in the absence of any signs of immunodeficiency with a viral load below 10^4 ^copies per ml plasma when analysed. Our data demonstrate significantly higher amounts of A3G and A3F in PBMC (Figure 3A and 3B), CD4+ T-cells (Figure 3C and 3D) and in peripheral lymph node cells (Figure 3G and 3H) of LTNPs compared with progressor macaques. For CD14+ monocytes, the difference in the APOBEC3 mRNA expression between LTNPs and progressors was only significant for A3F (Figure 3F), but not for A3G (Figure 3E). From a limited number of animals, we had sufficient material to perform Western blot analysis of A3G protein. Compared with uninfected control animals, A3G expression in PBMC was strongly increased in LTNP (additional file 2). Unfortunately, available antibodies showed no cross-reactivity with rhesus monkey A3F.

Taken together, the negative correlation between APOBEC3 levels and viral load as well as the high APOBEC3 levels found in LTNPs, suggest a positive influence of APOBEC3 on the disease course.

### Positive correlation of ISG mRNA levels with viral load and disease course

The increased expression of APOBEC3 in all cell types investigated in asymptomatic animals, suggests a regulation by infection specific factors. This is corroborated by a coordinated expression of A3G and A3F, which was observed in PBMC (p = 0.02), CD4+ T-cells (p = 0.02), peripheral lymph nodes (p = 0.003) in SIV-infected macaques. Possible candidates for this effect are interferons, as they play an important role during viral infections. In addition, IFN-α has been shown to induce A3G expression in human leukocytes through interferon response elements (ISRE) in the A3G promotor [15,37]. Similarly, we observed an IFN-α-induced, dose dependent increase of A3G and A3F transcription in simian PBMC *in vitro *(data not shown).

**Figure 4:**
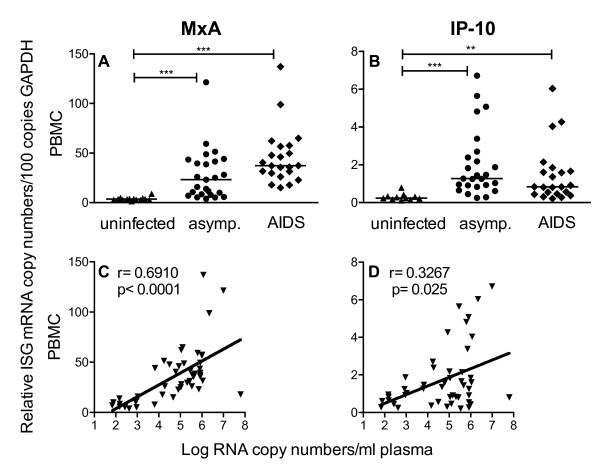
**mRNA levels of interferon-stimulated genes (ISGs) in uninfected and SIV-infected macaques**. Upper panels show relative MxA (A) and IP-10 (B) mRNA levels in PBMC of uninfected (triangles) and SIV-infected animals with (diamonds) or without (circles) AIDS symptoms. Each data point represents one individual animal. Relative ISG transcription levels are shown in copy numbers per 100 copies of GAPDH. Horizontal lines within the clusters are depicting the median. P values were calculated using the Kruskal-Wallis test with Dunn's multiple comparison analysis (**p < 0.001; ***p < 0.0001). Lower panels display the relationship between MxA (C) and IP-10 mRNA levels (D) and plasma viral load in PBMC. Viral load is depicted as log-transformed RNA copies per millilitre (ml) plasma. asymp., asymptomatic r; Spearman's correlation coefficient; line shows nonlinear regression p, P value.

**Figure 5:**
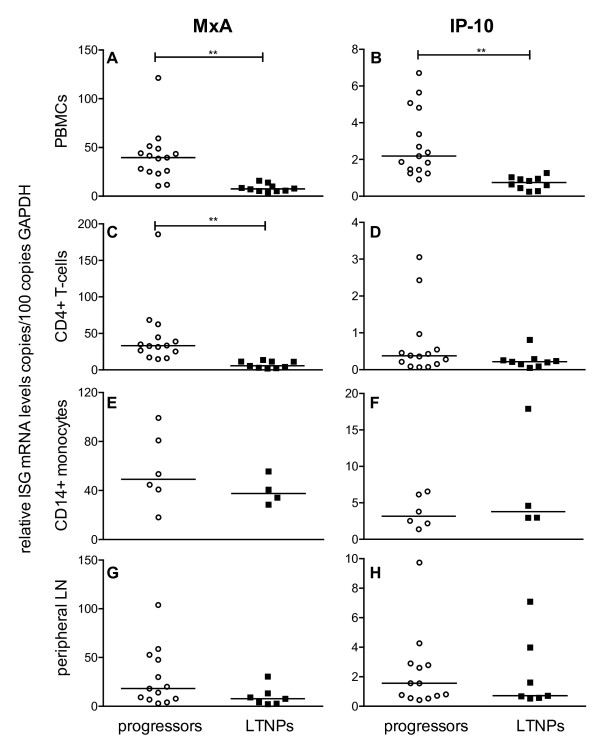
**mRNA levels of ISG in animals with different disease progression**. MxA (left panels) and IP-10 (right panels) mRNA levels were determined in SIV-infected progressors (open circles) or LTNP (squares). Relative ISG mRNA levels are shown in copy numbers per 100 copies of GAPDH in PBMC (A, B), CD4+ T-cells (C, D), CD14+ monocytes (E, F) and lymphocytes from peripheral lymph nodes (G, H). Each data point represents one individual animal. Horizontal lines within the clusters depicting are the median. Group differences were calculated using the Mann-Whitney test (**p < 0.001).

In order to investigate a potential influence of interferons on A3G and A3F levels *in vivo*, we quantified transcription levels of two ISGs, MxA (myxovirus resistance 1) and IP-10/CXCL10 (interferon-induced protein 10 kDa) as they represent conventionally used surrogate markers for interferon-mediated effects. Similar to A3G and A3F, we found a significant increase in the MxA and IP-10 mRNA levels in the PBMC of asymptomatic SIV-infected macaques compared with uninfected animals. In macaques with AIDS, MxA transcription levels were even higher than in asymptomatic monkeys, although not reaching significance (Figure 4A). IP-10 levels of all infected animals also remained above those of uninfected macaques (Figure 4B). This is in contrast to the results for A3G and A3F, where transcription rates in PBMC were comparable between animals with AIDS and uninfected controls (Figure 1A and 1B). MxA- and IP-10-expression in SIV-infected animals was also elevated in CD4+ T-cells, CD14+ monocytes and in both types of lymph nodes (data not shown). In contrast to expression levels of A3G and A3F, which negatively correlated with viral load, we found a positive correlation between MxA or IP-10 mRNA levels and viral load. Such an association was seen for both ISGs in PBMC (Figure 4C and 4D), in CD4+ T-cells and in peripheral as well as mesenteric lymph node cells, but only for MxA in CD14+ monocytes (data not shown). By dissecting MxA and IP-10 transcription of chronically infected rhesus monkeys into those of progressors and LTNPs, we observed lower mRNA levels of both ISGs in PBMC (Figure 5A and 5B) and lower MxA levels in CD4+ T-cells (Figure 5C) of LTNPs. Regarding the IP-10 mRNA levels in the CD4+ T-cells, there was no significant difference between progressors and LTNPs (Figure 5D). This was also true for the ISGs mRNA levels in CD14+ monocytes and in peripheral lymph nodes (Figure 5E-H).

These results, however, contrast the high A3G and A3F mRNA levels found in LTNPs (Figure 3). Together with the opposite correlations between plasma viral load and APOBEC3 and ISGs mRNA levels respectively, this may indicate a different regulation of A3G and A3F than the prototype ISGs during asymptomatic phase.

### A3G and MxA expression are increased during early SIV-infection

**Figure 6:**
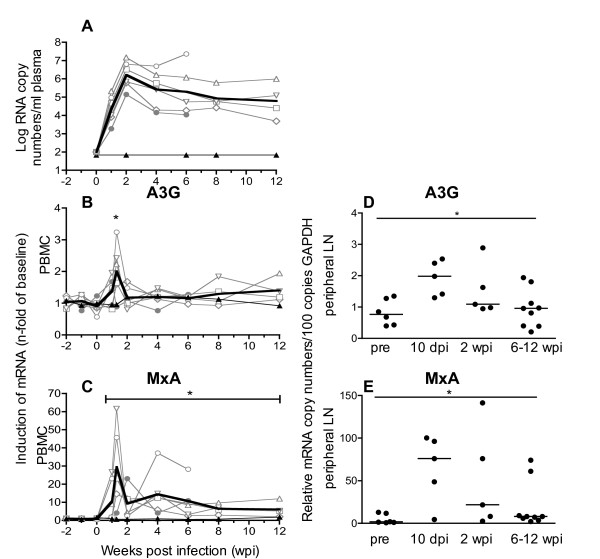
**Kinetics of RNA plasma viral load and mRNA levels of A3G and MxA in SIV-infected macaques**. Plasma viral loads as well as A3G and MxA mRNA levels in PBMC were determined longitudinally in seven macaques before and after inoculation with SIV (left panels A-C). Viral load is depicted as log-transformed RNA copies per millilitre (ml) plasma (A). Relative A3G (B) mRNA and relative MxA mRNA (C) in PBMC were calculated as copy numbers per 100 copies of GAPDH. Data are expressed as fold increase over baseline after normalization to the mean of three preinfection values. Fine grey lines with symbols represent individual infected animals. Fine black lines with triangles depict the one animal inoculated but not infected. Bold lines show mean values of infected animals. The asterisks indicate a significant difference to the mean of the three preinfection values calculated by Mann-Whitney test (*p < 0.05). Right panels show relative mRNA levels of A3G (D) and MxA (E) in lymphocytes isolated from peripheral lymph nodes at selected time points. Each data point represents one individual animal with horizontal lines showing the median. For comparison the data from 10 dpi and 2 wpi were combined. Statistical analysis was calculated using the Kruskal-Wallis test with Dunn's multiple comparison test (*p < 0.05). pre, preinfection values; dpi, days post infection; wpi, weeks post infection.

Our results from the cross-sectional study suggest an induction of A3G and A3F during the asymptomatic phase of infection. Therefore, we followed the time course of A3G and MxA levels during early infection. Seven animals were inoculated with different doses of SIV as part of an *in vivo *titration study. All macaques, except one of those inoculated with the lowest dose, became infected and showed a typical course of plasma viral load (Figure 6A). As shown previously, the inoculation dose did not influence viral replication kinetics *in vivo *[38]. This experiment was terminated early after infection and animals were euthanized at predetermined time points between six and 30 weeks post infection without signs of AIDS. Figure 6 shows the kinetics of A3G (B) and MxA transcription (C) for PBMC in these macaques normalized to the mean of three independently measured preinfection values. The inoculated macaque that remained uninfected served as control. Starting one week after infection, we observed a simultaneous increase of A3G and MxA transcripts in PBMC compared with preinfection values, which reached a maximum at ten days post infection (Figure 6B and 6C). This was shortly before peak viremia, which occurred at two weeks after infection (Figure 6A). These variations were not seen in the single animal that remained uninfected after inoculation. After a nadir at two weeks post infection, the MxA mRNA levels slightly increased again and remained significantly elevated above preinfection values (Figure 6C). Similarly, A3G mRNA decreased at two weeks post infection to levels only marginally above baseline, with a tendency to a slow increase thereafter (Figure 6B). Due to the limited number of animals, this rise, however, did not reach significance in the observation period of this experiment.

For some of the animals, it was also possible to quantify A3G and MxA mRNA at certain time points after SIV infection (either ten days or two weeks and six or 12 weeks post infection) in peripheral lymph nodes. By including available preinfection data from some of the animals, it was possible to illustrate a kinetic for the peripheral lymph nodes as well (Figure 6D and 6E). Similar to PBMC, we found significantly increased mRNA levels of A3G and MxA during the acute phase, at ten days post SIV infection. Later in the early asymptomatic phase, six to 12 weeks post SIV infection A3G and MxA mRNA levels were reduced to almost normal levels (Figure 6D and 6E).

### Disease stage specific regulation of APOBEC3 expression *in vivo*

**Figure 7:**
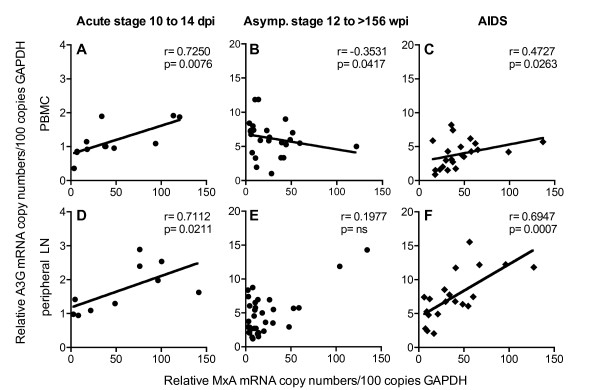
**Relationship between A3G and MxA mRNA levels during SIV disease stages**. Relationship between relative A3G and MxA mRNA levels in PBMC (A-C) and in peripheral lymph nodes (D-F) of SIV-infected asymptomatic macaques during acute infection (A and D, 10 to 14 dpi), in SIV-infected asymptomatic macaques during chronic infection (B and E, 12 to >156 wpi) and in SIV-infected macaques with AIDS (C and F). Relative mRNA levels are depicted as copy numbers per 100 copies of GAPDH. Each data point represents one SIV-infected animal. asymp., asymptomatic; r, Spearman's correlation coefficient; line shows linear regression; p, P value.

The parallel kinetics of A3G and MxA expression suggest common regulatory mechanisms for both genes at early stages of infection. Indeed A3G and MxA transcription was directly correlated in both PBMC and peripheral lymph nodes during the acute phase (ten to 14 days post infection) (Figure 7A and 7D). This contrasts the results at later time points (12 to >156 weeks post infection) in asymptomatic SIV-infected macaques, showing no or even a negative correlation between MxA and A3G mRNA level in PBMC and peripheral lymph nodes (Figure 7B and 7E) or CD4+ T-cells (p = 0.132). Such negative relationship was also found between IP-10 and A3G in PBMC in the asymptomatic phase (p = 0.04). Similar results were seen when comparing A3F with ISGs in all cell types investigated from asymptomatic animals (data not shown). Interestingly in macaques with signs of AIDS, levels of MxA and A3G (Figure 7C and 7F) or A3F were again positively correlated in PBMC (p = 0.028) and peripheral lymph nodes (p = 0.038). Such positive correlations were also observed between IP-10 and A3G (p = 0.012 PBMC; p < 0.0001 peripheral lymph nodes) or A3F (p = 0.04 PBMC; p = 0.044 peripheral lymph nodes) at the time of necropsy.

These data suggest that several factors are involved in the regulation of A3G and A3F during SIV infection. The relative contribution of these different mechanisms seems to vary with the stage of infection.

## Discussion

Members of the APOBEC family of deaminases, such as A3G and A3F, have been described as potent retrovirus restriction factors, capable of inhibiting replication of several viruses including HIV-1 *in vitro *[4,39-41]. In rodents, it has been clearly demonstrated that APOBEC3 contributes to restriction of Friend MuLV infection *in vivo *[42,43]. The role of APOBEC3 in the pathogenesis of AIDS, however, is still under debate [24,25,27,44,45]. Therefore, we determined A3G and A3F transcription in SIV-infected rhesus macaques and linked it to plasma viral load and disease progression.

Compared with uninfected control animals, we found increased mRNA levels of both A3G and A3F in SIV-infected monkeys during the asymptomatic phase of the disease. So far, several studies have investigated A3G transcription in HIV-infected subjects, however with inconsistent results [25-27,44,46,47]. Whereas some reported increased levels of A3G in HIV-infected subjects [25,44], others found lower A3G mRNA compared with uninfected individuals [27,47]. In the few studies on A3F, similarly discrepant results were observed [25,27]. Some of the inconsistencies might be attributed to methodological differences as both fresh and cryopreserved cells with or without polyclonal stimulation were used. However, the wide variation between infected individuals, also seen in our study, suggests that the most important source of the conflicting data is likely differences in the selection of patients with regard to disease stage and viral load. In the present study, animals that had progressed to AIDS had lower A3G and A3F levels in blood than asymptomatic macaques. Although an association between disease stage in humans and APOBEC3 transcription has not been investigated explicitly, this is corroborated by recent findings of very low A3G mRNA levels in patients with CD4 counts below 200 [26]. On the other extreme, as reported for HIV-patients [46], we observed the highest mRNA levels among asymptomatic animals in a group of LTNPs, representing animals that had survived for more than three years after infection. This association between APOBEC3 mRNA and disease progression is substantiated by our finding of a negative correlation between APOBEC3 levels and plasma viral load. These results are in agreement with several previous reports, which documented higher A3G [25,26,46] and A3F [25] mRNA levels in PBMC of patients with lower viral load. By contrast, another study did not find a correlation between APOBEC3 mRNA and viral load [27]. As observed previously [26], APOBEC3 mRNA copies in individuals with high viral load were often below the levels of uninfected controls. These findings may explain the decreased A3G levels in PBMC of infected compared with uninfected individuals reported by some studies, probably due to differences in the composition of the patient groups.

Our study is the first one, which quantified APOBEC3 mRNA in the context of an immunodeficiency virus infection not only in whole PBMC, but also in leukocyte subsets susceptible to infection such as CD4+ T-cells and CD14+ monocytes as well as in the lymph nodes as major virus replication sites. Despite slight variations, the same tendencies were observed. A3G and A3F mRNA levels correlated negatively with viral load and the highest values were observed in LTNPs. Interestingly, the dichotomy in A3G transcription of individuals with higher or with lower A3G levels than uninfected controls was more pronounced in CD4+ T-cells possibly due to differences in the cellular composition. In general, mRNA levels determined in PBMC mirrored the situation in CD4+ T-cells or monocytes. However, this does not reflect the conditions in lymph nodes. In lymph nodes from two different anatomical regions, we also detected high amounts of A3G and A3F mRNA in LTNPs. In contrast to blood however, APOBEC3 levels in lymph nodes were still increased in animals with AIDS. This might be explained by the fact that AIDS-associated immunological alterations occur later in lymphatic tissues than in blood [48,49]. Since we sacrificed the animals when first signs of AIDS appeared, the situation in lymph nodes may still be more similar to the asymptomatic phase. Also differences in the cellular composition or in the microenvironment between the different compartments [50] could be responsible as APOBEC3 transcription varies between different cell types and tissues [21,51,52] and can be influenced by interferons, cytokines and chemokines [17,19,20,22,37,51]. To our knowledge, APOBEC3 mRNA in lymph nodes of HIV-patients has yet not been studied to substantiate our results with findings from the human setting.

Our data clearly demonstrate a negative correlation of A3G and A3F mRNA levels with viral load and an association of high A3G and A3F mRNA levels with prolonged survival, suggesting a possible effect of APOBEC3 on viral replication *in vivo *favorably influencing disease progression. It has been hypothesized, that the higher A3G levels in LTNPs or patients with low viral load are due to preexisting differences in gene expression [45]. However, our longitudinal studies suggest that A3G transcription is actually up-regulated after infection. This is in line with the results of a recent publication where A3G transcription was compared in the same individuals before and after infection [25]. In addition, we found coordinated transcription of A3G and A3F in all cell populations investigated only in SIV-infected animals, which points to common regulatory mechanisms induced by the infection. Interestingly, increased A3G levels have also been reported in patients with HCV-infection [53]. General to viral infections is a potent induction of type one interferons and several studies have demonstrated stimulation of A3G and A3F expression by interferons *in vitro *[15,37,54,55]. Therefore, we have determined the transcription of conventionally used prototype interferon-stimulated genes to assess interferon activity in our animals. As reported previously for both HIV-infected humans and SIV-infected macaques [56-58], we observed an early and strong rise of MxA mRNA, which reached its maximum ten days after infection, hence preceding peak viral load. This up-regulation of ISG was paralleled by a more moderate increase in A3G mRNA levels. With resolution of peak viremia both MxA and A3G levels declined but remained slightly above preinfection levels in both PBMC and lymph node cells. These parallel kinetics indicate that APOBEC3 deaminases are downstream effector molecules of a type I interferon response in acute viral infections. In the chronic phase of SIV-infection, however, we found a divergent expression of prototype ISG and A3G/A3F. Whereas MxA and IP-10 transcription increased with higher viral replication, A3G and A3F mRNA levels were negatively correlated with plasma viral load in SIV-infected asymptomatic animals. Thus, other mechanisms must be responsible for the higher APOBEC3 levels found in asymptomatic animals, especially in LTNPs. As acute phase and end stage disease are characterized by a lack of virus specific immunity and LTNPs show a better specific immune response than progressors [59], mediators associated with a potent immune reaction are likely candidates for this additional APOBEC3 modulation in asymptomatic animals. Cytokines like IL-2 and IL-15 have been shown to induce A3G expression *in vitro *[19,22], PBMC from LTNPs produce higher amounts of IL-2 [60] and adjuvanting vaccine vectors with IL-15 improves protection against pathogenic challenge in the SIV/macaque model, partly through an induction of A3G [22]. In this context it would be interesting to explore the effect of HAART on APOBEC3 levels as effective antiretroviral treatment has been shown to restore IL-2 production by antigen specific cells [61] and IL-15 levels correlated with better outcome of structured treatment interruption [62]. In addition, it has been reported that cross-talk between dendritic cells and T-cells also results in the induction of A3G expression by both contact dependant mechanisms via CD40-CD40L interaction and through soluble mediators such as IL-15 [17]. Dendritic cells are depleted in progressive HIV-infection [63], but accumulate in lymph nodes of LTNPs exhibiting elevated CD40 expression [64]. Increased signaling through CD40 ligand may thus be responsible for the higher A3G expression in the asymptomatic phase. Consistent with the reported loss of CD40 expressing dendritic cells in AIDS patients [64], this regulatory pathway attenuates leading to the lower APOBEC3 transcription, which we observed in PBMC of animals with symptoms of AIDS. At this stage of the infection, A3G and A3F mRNA levels once again seem to be governed solely by interferons as they correlated with the transcription of ISGs.

An alternative explanation for the disease stage specific relationship between APOBEC3 and ISG expression could be shifts in the composition of cells. Some groups have reported a stronger induction of A3G and A3F transcription by IFN-alpha for macrophages compared with CD4+ T-cells [21,51,55] and for resting compared with activated CD4+ T-cells [15]. However, as we see the same disease stage specific pattern for all cell types investigated, such differences in sensitivity to interferon stimulation probably do not contribute to the discrepancy between APOBEC3 and ISG transcription in asymptomatic animals.

In summary, we think that increased amounts of interferons in SIV-infected macaques influence APOBEC3 mRNA levels throughout the infection. However, as evidenced by the much lower induction of A3G during peak viremia compared with prototype ISG, the effect of interferons on the transcriptional control of APOBEC3 deaminases *in vivo *is moderate. This interferon-mediated basic regulation is then overlaid by additional modulatory mechanisms, which need time to build up and are strongest in LTNPs, but ultimately vanish during AIDS. Such mechanisms may also potentially become activated in uninfected individuals as various vaccination regimens can induce a long lasting upregulation of A3G in macaques [22,65]. In addition, several studies have documented increased A3G mRNA levels in HIV-1 exposed but seronegative individuals [26,44], who are known to mount a subtle HIV-specific cellular immune response [66]. Interestingly, cessation of exposure was associated with both decreased A3G levels [26] and with a loss of anti-HIV-1 T-cell response [67]. Exploring the mechanisms leading to increased APOBEC3 levels in LTNPs or HIV-1 exposed but seronegative individuals should provide useful information for new therapeutic approaches.

The results from this and previous studies suggest that increased APOBEC3 mRNA levels are associated with lower viral load and slower disease progression. According to our data, A3G and A3F mRNA levels are increased also in CD4+ lymphocytes and CD14+ monocytes, the cellular targets of HIV, and in lymph nodes where the majority of CD4+ T-cells resides and where abundant viral replication takes place. Information on other tissues with high viral load such as mucosa associated lymphoid tissue is sparse. So far, only cervical biopsies have been investigated where A3G levels were comparable between infected and uninfected individuals [44].

Evidence that APOBEC3 actually exerts its deaminase activity *in vivo *is derived from studies, which found the typical G to A mutations imprinted on the viral genomes in HIV-infected patients [12,26,68]. The extent of these characteristic mutations correlated with A3G transcription [26], indicating that increased APOBEC3 expression may overcome the restraints imposed by Vif [2]. Moreover, higher hypermutation rates conforming to the A3G and A3F sequence preferences were associated with lower viral load and higher CD4 cell counts [12,68]. On the other hand, increased A3G/A3F expression may be beneficial even without demonstrable hypermutations as APOBEC3 enzymes can restrict viral replication through mechanism distinct from cytidine deamination [10,11,69].

## Conclusions

By demonstrating an association between increased A3G and A3F mRNA levels with prolonged survival in the defined experimental setting of an animal model, we provide further evidence for a potential protective role of APOBEC3 in the pathogenesis of HIV-infection. Depending on the stage of the infection, several mechanisms seem to contribute to the up-regulation of APOBEC3 transcription *in vivo*. We postulate that A3G is regulated in vivo by type I interferons, an effect which appears to dominate in the acute and AIDS phases. In addition, there are other regulatory mechanisms, most strongly present in LTNP, which govern APOBEC regulation in the asymptomatic stage. Future investigations to elucidate the regulatory mechanism may help to exploit these intrinsic antiretroviral factors for anti-HIV therapy and vaccination.

## Materials and methods

### Animals

Rhesus macaques (*Macaca mulatta*) of Indian origin were housed at the German Primate Centre under standard conditions according to the German animal protection law which complies with the European Union guidelines on the use of non-human primates for biomedical research. Animals were infected either via the tonsils with SIVmac239 [70] or intravenously with a SIVmac251-derived virus stock [71], both prepared in rhesus monkey peripheral blood mononuclear cells. Sampling of blood was carried out under ketamine anesthesia. For sampling of lymph nodes, the animals were anesthetized with a mixture of 5 mg ketaminhydrochloride, 1 mg xylazinhydrochloride and 0.01 mg atropine sulfate per kg body weight. Mesenteric lymph nodes were obtained on the day of necropsy.

In a cross-sectional study, 53 infected animals were grouped according to their clinical stage. Twenty-nine macaques, which were clinically asymptomatic, were investigated during the chronic phase of the infection. Some of these were sacrificed according to the experimental schedule without signs of AIDS. 24 macaques were euthanized when first signs of AIDS appeared, i.e. anorexia, incurable diarrhoea, *Pneumocystis jirovecii *infection or neurological dysfunction as judged from clinical as well as necropsy and histopathological findings, which were available for each macaque (additional file 3).

Blood and/or lymph node samples were obtained at different time points after infection ranging from 12 to >156 weeks post infection (wpi) for SIV-infected asymptomatic macaques (median 62 wpi) and 22 to 138 wpi for animals with AIDS (median 80 wpi). Ten of the asymptomatic animals survived with a set point viremia of below 10^4 ^RNA copies/ml plasma for more than three years and were thus regarded as LTNP. The remaining asymptomatic animals had viral loads above 10^4 ^RNA copies/ml plasma at the time of investigation and later showed a progressive disease course leading to AIDS within three years of infection.

Equal numbers of animals had been infected with SIVmac239 or SIVmac251 and mRNA levels of A3G, A3F, MxA and IP-10 were not influenced by the infecting virus strain. 12 uninfected clinically healthy rhesus macaques were used as negative control group.

In a longitudinal study, two animals were inoculated intravenously with 100 TCID50, three animals with 10 TCID50 and two animals with 1 TCID50 of SIVmac251 as part of an *in vivo *titration experiment aimed at defining the in vivo infective dose of a new monkey PBMC-derived virus stock. This SIVmac251 challenge virus was prepared on PHA-stimulated PBMC from several monkeys using SIVmac251 [71] as inoculum. Supernatants were harvested and pooled. After filtration, aliquots were prepared and stored at -140C and the TCID50 was determined on C8166 cells.

### Lymphocyte isolation

Peripheral blood was collected by venipuncture and peripheral blood mononuclear cells (PBMC) were isolated via ficoll-paque gradient centrifugation (lymphocyte separation medium, PAA laboratories, Pasching, Austria). PBMC were washed with phosphate-buffered saline (PBS). CD4+ T-cells and CD14+ monocytes were enriched from fresh PBMC by positive selection using magnetic beads (Miltenyi Biotec, Bergisch-Gladbach, Germany) and monoclonal antibodies to either CD4 or CD14. The purity of the isolated CD4+ T-cells and CD14+ monocytes were analyzed by flow cytometry on a LSR II flow cytometer (Becton Dickinson, Heidelberg, Germany) with the following fluorescence conjugated antibodies: anti-CD3 Alexa 700 (BD Biosciences); anti-CD4 Alexa 405 (BD Biosciences), anti-CD14 PerCP Cy5.5 (BD Biosciences), anti-CD20 PECy 7 (BD Biosciences) and anti-CD45 FITC (Mitenyi Biotec). Only CD4+ T-cells and CD14+ monocytes with purity above 90% were used for downstream applications. Tissue cell suspensions from lymph nodes were prepared by dissecting the lymph nodes with scalpels and forceps in RPMI 1640 (PAN Biotech, Aidenbach, Germany) supplemented with 10% FCS (PAN Biotech, Aidenbach, Germany), 100 U/ml penicillin (PAN Biotech, Aidenbach, Germany) and 100 μg/ml streptomycin (PAN Biotech, Aidenbach, Germany) (complete RPMI) and passing the homogenate through a cell strainer (100 μm nylon; BD Biosciences, Heidelberg, Germany). Separated cells were washed twice in PBS and living cells were counted by trypan blue exclusion.

### RNA isolation and cDNA synthesis

Total cellular RNA was isolated from 2 × 10^6 ^to 5 × 10^6 ^cells with the RNeasy Plus Mini Kit (Qiagen, Hilden, Germany), according to the manufacturer's instructions. Purified RNA was quantified by measuring the optical density at 260 nm (OD_260_). All samples had an OD_260_/OD_280 _ratio of 1.9 or greater. The quality of the isolated RNA was randomly checked by Agilent 2100 Bioanalyzer (Agilent Technologies, Böblingen, Germany) featuring RIN values (RNA Integrity Number) of at least 8.0. Synthesis of cDNA was carried out with random hexamer primers and SuperScript III First-Strand Synthesis System for RT-PCR kit (Invitrogen GmbH, Karlsruhe, Germany), according to the manufacturer's protocol.

### Quantification of A3F, A3G, MxA and IP-10/CXCL10 mRNA

We established a real-time PCR assay to quantify A3G, A3F, MxA and IP-10/CXCL10 mRNA levels in lymphocytes/leukocytes using SYBR Green (Qiagen, Hilden, Germany) chemistry with primers designed to uniquely amplify A3G (Genbank accession number AY331716), A3F (Genbank accession number NM_001042373), MxA (Genbank accession number EF101561) and IP-10/CXCL10 (Genbank accession number AY044446). The following primers (Sigma, Hamburg, Germany) were used: A3G forward, 5'-TCTACGCAACCAGGCTCCA-3' (nt 702 to 720); A3G reverse 5'-GGAATCAGGTCCAGGAAGCA-3' (nt 779 to 760); A3F forward, 5'-CAGTAATGTGAAGCTCGCCATC-3' (nucleotides [nt] 882 to 903); A3F reverse, 5'-TGCTGGTAATGTGTATCCCAGAA-3' (nt 947 to 925); MxA forward, 5'-AGGAGTTGCCCTTCCCAGA-3' (nt 295 to 313); MxA reverse, 5'-TCGTTCACAAGTTTCTTCAGTTTCA-3' (nt 372 to 348); IP-10/CXCL10 forward, 5'-GATTTGCTGCCTTGTCTTTCTGA-3' (nt 21 to 43); IP-10/CXCL10 reverse, 5'-CAGGTACAGCGTACAGTTCTTGAGA-3' (nt 95 to 71). Primers were selected in less conserved regions to limit sequence homologies with other APOBEC3 genes. Later, significant homology was detected in the A3F primer region with A3D, a sequence which was not available at the beginning of the study. Sequences for the MxA primers were taken from Abel *et al*. [72]. Glyceraldehyde-3-phosphate dehydrogenase (GAPDH) (Genbank accession number XM_001105471) was used as a house keeping gene with the following primers taken from Rodriguez-Jimenez et al. forward, 5'-CCTGCACCACCAACTGCTTA-3'(nt 525 to 544); reverse, 5'-CATGAGTCCTTCCACGATACCA-3' (nt 598 to 577) [73]. The reactions were performed in Micro Amp optical tubes or plates (Applied Biosystems GmbH, Darmstadt). Each 25 μl reaction mixture contained 12.5 μl 2 × QuantiTect SYBR Green PCR master mix (Qiagen, Hilden, Germany), 1 μl of each 10 μM primer, and 2 μl cDNA products. The reactions were run in an ABI Prism 7500 with one cycle at 95°C (15 min) followed by 40 cycles at 95°C (15 s) and 55°C (1 min). Validation experiments were performed to determine the specificity and efficiency of the primers to selectively amplify the target gene. Melting curves and agarose gel documentation demonstrated the existence of a single product (additional file 4). The calculated efficiency for all primers, determined by dilution experiments, was from 97% to 99 %, thus target sequences were amplified with similar efficiencies. All samples were run at least in duplicates. The results were analyzed by Sequence Detection Software (Applied Biosystems GmbH, Darmstadt), and A3F, A3G, MxA and IP-10/CXCL10 mRNA levels were calculated as copy numbers relative to 100 copies of GAPDH.

### Western blot for APOBEC3G protein

PBMCs were lysed with ice-cold buffer containing 50 mM 4-(2-hydroxyethyl)-1-piperazineethanesulfonic acid (HEPES), pH 7.4, 150 mM NaCl, 0.1% octyl phenoxylpolyethoxylethanol (Nonidet-P40), 0.5 mM phenylmethanesulfonylfluoride (PMSF), 1% protease inhibitor cocktail (Sigma, Hamburg, Germany). Proteins were quantified by a bicinchoninic acid protein assay reagent kit (Pierce, Rockford, USA). An equal amount of protein (20 μg) from different animal samples was loaded in individual lanes of a 12% SDS-polyacrylamide gel. After electrophoretic separation, the proteins were transferred to nitrocellulose membrane (Schleicher & Schuell Bioscience, UK). Membranes were blocked with 5% milk powder phosphate-buffered saline with 0.1% Tween-20 and probed with monoclonal anti-APOBEC3G at 1:1000 (Immunodiagnostics, Woburn, MA, USA) at 4°C overnight. Membranes were washed with phosphate-buffered saline with 0.1% Tween-20 three times for 5 minutes and incubated for one hour with secondary antibody conjugated with horseradish proxidase (Jackson ImmunoResearch, Suffolk, UK) and detected by chemiluminescence (Super Signal West Pico Chemoluminescence Kit Pierce, Rockford, USA).

### Quantification of Plasma viral RNA and cell associated viral load

Isolation of viral RNA was performed from plasma samples according to the MagAttract Virus Mini M48 protocol (Qiagen, Hilden, Germany). Purified SIV RNA was quantified with TaqMan-based real-time PCR on an ABI-Prism 7500 sequence detection system (Applied Biosystems GmbH, Darmstadt) as described [74]. Amplified viral RNA was calculated as SIV-RNA copies per millilitre plasma.

Cell associated viral loads in organs were determined by limiting dilution coculture of monkey PBMC and the permanent T-cell C8166 as indicator cells, which were adhered to concanavalin A-coated microtiter plates. Viral replication in cultures was visualized by immunoperoxidase staining of intracellular antigen [50].

### Statistics

The statistical analyses were calculated with GraphPad Prism version 5 (GraphPad software). For interpretation between more than two groups the Kruskal-Wallis test with Dunn's multiple comparison analysis was used and for comparison between two groups the nonparametric two tailed Mann-Whitney's U test were used. For correlation the nonparametric two tailed Spearman test was performed. Significance level was always set at p-values less than 0.05.

## Competing interests

The authors declare that they have no competing interests.

## Authors' contributions

BM carried out the experiments analyzed the data and drafted the manuscript. US determined the viral load and participated in the data analysis. DM participated in the design of the study. CSH determined cell associated viral load and participated in the data analysis. SS conceived of the study, participated in its design and coordination and helped to draft the manuscript. All authors contributed to revision of the draft manuscript and approved the final version.

## Supplementary Material

Additional file 1 **Correlation of cell associated viral load in LN with plasma viral load and LN A3G mRNA levels**. Two figures depicting a significant association between cell associatiated viral load in LNmes and viral RNA levels in plasma and A3G mRNA levels in LNmes respectively. Click here for file

Additional file 2 **Western blot of A3G protein**. Western blot analysis of PBMC shows higher levels of A3G protein in LTNP than uninfected animals. Click here for file

Additional file 3 **Major clinical and pathological findings in animals with AIDS**. Table listing major clinical and pathological findings in individual animals. Click here for file

Additional file 4 **Melting curves and gel electrophoresis of A3G and A3F PCR products**. Melting curves and gel documentation shows single products of PCR reactions with A3G and A3F primers. Click here for file
