# Supplementary material for: Increased APOBEC3G and APOBEC3F expression is associated with low viral load and prolonged survival in simian immunodeficiency virus infected rhesus monkeys
Source: Retrovirology. 2011 Sep 28;8:77. doi: 10.1186/1742-4690-8-77 (PMC3192745; doi:10.1186/1742-4690-8-77)
Supplement: Additional file 1 — Correlation of cell associated viral load in LN with plasma viral load and LN A3G mRNA levels. Two figures depicting a significant association between cell associatiated viral load in LNmes and viral RNA levels in plasma and A3G mRNA levels in LNmes respectively. [file 1742-4690-8-77-S1.PDF]

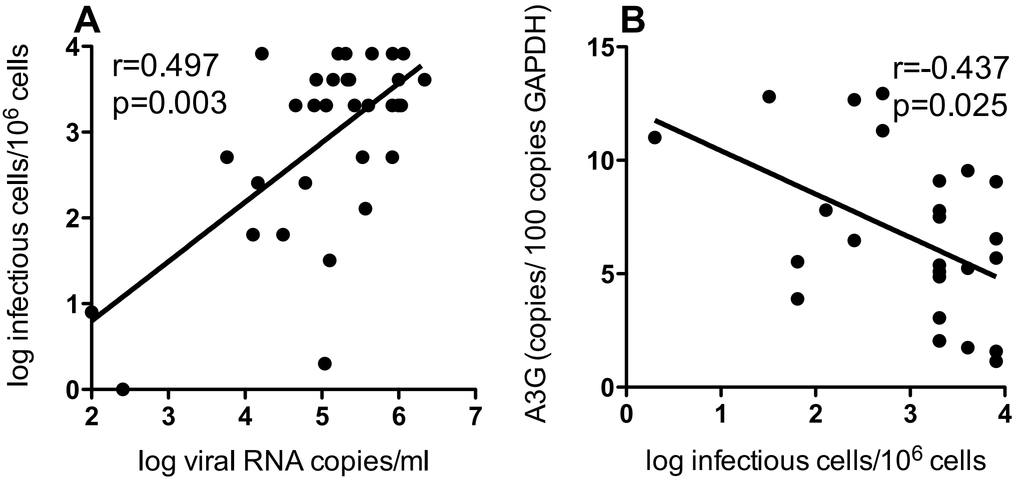

Suppl. Fig. 1. Relation of cell associated VL in LNmes and plasma VL and A3G levels in LNmes.

Cell associated viral load in LNmes, depicted as log transformed number of infectious cells per 10^6 LNmes cells, was correlated with plasma viral load (A) and A3G mRNA levels in LNmes (B). Viral load is depicted as log-transformed RNA copies per millilitre (ml) plasma. Relative APOBEC3 mRNA levels are shown in copy numbers per 100 copies of GAPDH in LNmes cells.r, Spearman's correlation coefficient; line shows nonlinear regression; p, P value;
