# Supplementary material for: Increased APOBEC3G and APOBEC3F expression is associated with low viral load and prolonged survival in simian immunodeficiency virus infected rhesus monkeys
Source: Retrovirology. 2011 Sep 28;8:77. doi: 10.1186/1742-4690-8-77 (PMC3192745; doi:10.1186/1742-4690-8-77)
Supplement: Additional file 2 — Western blot of A3G protein. Western blot analysis of PBMC shows higher levels of A3G protein in LTNP than uninfected animals. [file 1742-4690-8-77-S2.PDF]

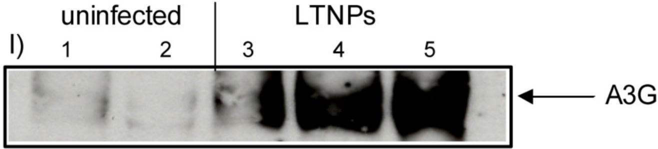

Suppl. Fig. 2. A3G protein expression in PBMC
Western blot analysis of A3G protein ~42kD in PBMC of two uninfected (lane 1 and 2) and three LTNPs (lane 4-5).
