# Supplementary material for: Increased APOBEC3G and APOBEC3F expression is associated with low viral load and prolonged survival in simian immunodeficiency virus infected rhesus monkeys
Source: Retrovirology. 2011 Sep 28;8:77. doi: 10.1186/1742-4690-8-77 (PMC3192745; doi:10.1186/1742-4690-8-77)
Supplement: Additional file 3 — Major clinical and pathological findings in animals with AIDS. Table listing major clinical and pathological findings in individual animals. [file 1742-4690-8-77-S3.PDF]

Suppl. Table 1: Major clinical and pathological findings in animals with AIDS

Animal number	Major clinical and pathological findings
2141	Abcessae, parasitic enteritis, interst. pneumonia
2161	Cryptosporidia in liver, systemic leukosis, parasitic enteritis
2165	PcP, parasitic enteritis
2187	SIV-vasculopathy, cardiac insufficiency
2188	malignant B-cell lymphoma, meningitis
2191	parasitic enteritis
2192	parasitic enteritis
2194	malignant lymphoma, parasitic enteritis
2208	PcP, parasitic enteritis
2250	Enteritis, diarrhia, meningitis
11612	PcP, parasitic enteritis, mycotic dermatitis
12056	parasitic enteritis, interst. pneunomia
12531	Mycobacteria, SIV-vasculopathy, interst. pneunomia
12534	Cryptosporidia in liver, interst. pneunomia
12544	systemic leukosis, parasitic enteritis
13248	parasitic enteritis
13249	PcP, parasitic enteritis
13252	PcP, parasitic enteritis, mycotic dermatitis
13253	paralysis of lower limbs, chronic enteritis
13256	parasitic enteritis, interst. Pneunomia
13258	parasitic Cholangitis, alveolar lung edema
13261	PcP, parasitic enteritis
13262	PcP, parasitic enteritis, encephalopathy

SIV-infected animals were constantly monitored and physical examinations were performed by experienced veterinarians in regular intervals. Animals were euthanized when their health status deteriorated developed clinical signs of AIDS like untreatable diarrhea, pneumonia, kachexia or paraplegia signs. Diagnosis of AIDS was confirmed by gross pathological examination during necropsy followed by histopathological assessment. Presence of pathogens was assessed by routine diagnostic methods.
PcP: Pneumocystis jirovecii pneumonia
