# Supplementary material for: Increased APOBEC3G and APOBEC3F expression is associated with low viral load and prolonged survival in simian immunodeficiency virus infected rhesus monkeys
Source: Retrovirology. 2011 Sep 28;8:77. doi: 10.1186/1742-4690-8-77 (PMC3192745; doi:10.1186/1742-4690-8-77)
Supplement: Additional file 4 — Melting curves and gel electrophoresis of A3G and A3F PCR products. Melting curves and gel documentation shows single products of PCR reactions with A3G and A3F primers. [file 1742-4690-8-77-S4.PDF]

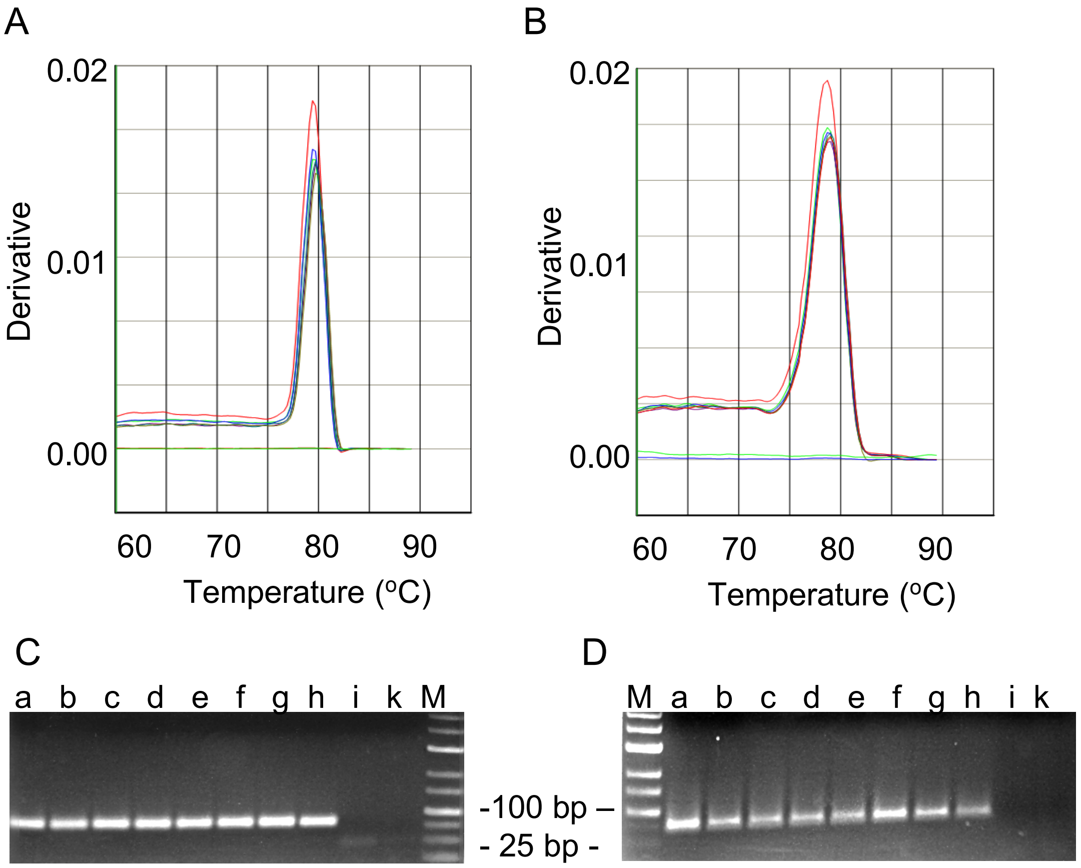

Suppl. Fig. 3: **Dissociation curves and gel documentation of APOBEC qPCR reaction products.**

Upper panels show dissociation curves of PCR-products generated with (A) APOEC3G and (B) APOBEC3F primer pairs and no-template controls. The first derivative of the rate of change in fluorescence as a function of temperature is shown. The Tm of the PCR-products is 82.0 ^{o}C for APOBEC3G and 78.9 ^{o}C for APOBEC3F whereas the no template controls show no fluorescence. Lower panels depict images of PCR-products generated with (C) APOBEC3G and (D) APOBEC3F-primers separated by electrophoresis on a 3 % agarose gel and stained with ethidium bromide. Lanes a-h: typical amplification products, i-k: no template control, M: DNA size marker (low range DNA ladder, Fermentas). The size in bp is shown for the 100 bp and 25 bp products).
